# Allergens from wheat and wheat products: A comprehensive review on allergy mechanisms and modifications

**DOI:** 10.1016/j.fochx.2025.102871

**Published:** 2025-08-05

**Authors:** Nan Jiang, Yu Wang, Yasai Sun, Zhe Gao, Dongcheng Liu, Bimal Chitrakar

**Affiliations:** a College of Food Science and Technology, Hebei Agricultural University, Baoding 071001, China; b State Key Laboratory of North China Crop Improvement and Regulation, College of Agronomy, Hebei Agricultural University, Baoding 071001, China

**Keywords:** Wheat, Allergens, Mechanism, Modification

## Abstract

Wheat is one of major human staple foods, contributing an important source of dietary protein. However, some people are allergic to wheat-based foods. They are accompanied by many symptoms, such as wheat dependent exercise induced anaphylaxis, atopic dermatitis, anaphylactic shock, which seriously affect the quality of life. Food processing technologies were proven affecting allergen allergenicity to varying degrees. Therefore, it is imperative to use effective methods to reduce wheat sensitization. This manuscript summarizes the mechanisms of wheat allergy, antigenic epitopes, cross-allergy with other cereals, and animal model studies. Then, the various modifications used to reduce wheat sensitization and their advantages and disadvantages are described. Among them, high hydrostatic pressure treatment has become a research hotspot because of its non-thermal in nature, giving better retention of nutritional value and sensory properties of the final product, and significantly reducing the allergenicity.

## Highlights

• Wheat allergy has caused significant dietary problems in allergic populations.
• Wheat allergens.
• High hydrostatic pressure technology can reduce allergenicity while retaining nutritional value and sensory properties.
• Bioinformatics prediction of epitopes can effectively assess potential sensitization of unknown allergens.
• Animal testing is an effective method for validating the results of desensitization research before application.

## 1 Introduction

Wheat (*Triticum aestivum* L.) is a cereal plant widely grown around the world with its global importance as one of the staple grains, which cannot be overstated. The production of wheat in 2024 was 792.2 million tons, which was about 27.8 % of the global cereal production (FAO, 2024). About 40 % of the global population depends on these sources for almost all of their daily energy needs. Wheat is nutritious and rich in starch, protein, lipids, minerals, vitamins, etc., and its nutritional value ranks among the top of the cereals, providing about 20 % of daily dietary calories and proteins, and the comparison of the nutritional composition of other cereals is shown in Table 1(U.S. Department of Agriculture, A. R. S., Beltsville Human Nutrition Research Center, 2025). However, wheat contains allergenic proteins which cause allergic reactions (Rao, Li, & Xue, 2023), and pose serious health hazards to some individuals.

**Table 1:** Proximate nutrients in 100 g of cereals.

Cereal Name		Wheat	White Rice	Corn	Millet	Barley	Oat	Sorghum
Energy		362 kcal	359 kcal	85 kcal	376 kcal	367 kcal	389 kcal	373 kcal
Water		11.1 g	11.6 g	80.2 g	10.2 g	9.9 g	8.86 g	10.6 g
Protein		12 g	6.94 g	2.79 g	10 g	8.72 g	13.2 g	10.1 g
Total lipid (fat)		1.7 g	1.3 g	1.63 g	4.19 g	2.45 g	6.31 g	4.22 g
Carbohydrates	Carbohydrate	74.6 g	79.8 g	14.7 g	74.4 g	77.4 g	69.9 g	73.6 g
	Fiber	3 g	0.5 g	2.4 g	2.6 g	12.8 g	10.5 g	8.3 g
Minerals	Calcium	22 mg	6 mg	1 mg	9 mg	36 mg	43 mg	15 mg
	Iron	1.18 mg	0.22 mg	0.39 mg	2.53 mg	3.3 mg	4 mg	3.9 mg
	Magnesium	36.1 mg	22.9 mg	25.8 mg	106 mg	88 mg	125 mg	136 mg
	Phosphorus	134 mg	94 mg	75 mg	258 mg	234 mg	372 mg	294 mg
	Potassium	150 mg	75 mg	237 mg	214 mg	367 mg	373 mg	367 mg
Vitamins	Thiamin	0.298 mg	0.09 mg	0.079 mg	0.411 mg	0.225 mg	0.39 mg	0.45 mg
	Niacin	1.59 mg	1.25 mg	1.59 mg	4.86 mg	5.94 mg	1.94 mg	6.1 mg
	Vitamin B-6	0.085 mg	0.052 mg	0.15 mg	0.192 mg	0.2 mg	0.148 mg	0.191 mg

**Fig. 1:**
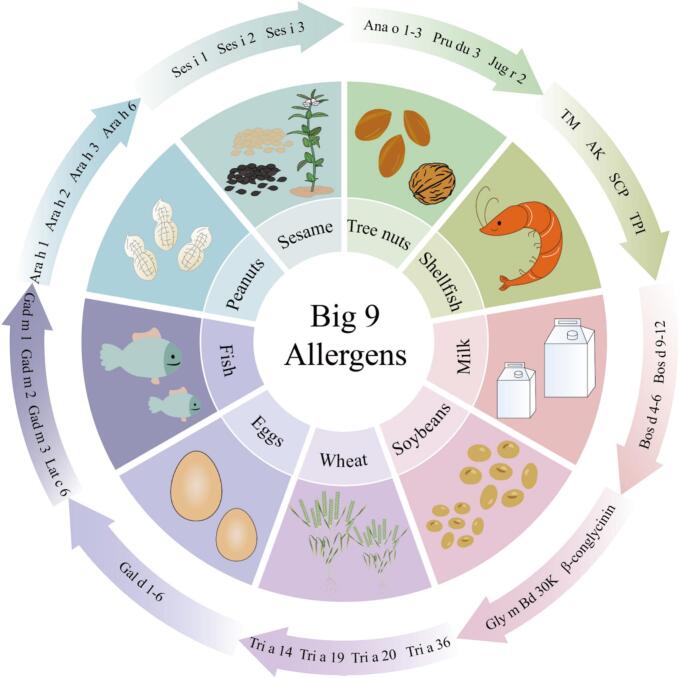
Big 9 allergens.

In 2023, the U.S. Food and Drug Administration (FDA) officially listed sesame as the ninth major food allergen. After that, milk, soybeans, wheat, tree nuts, peanuts, shellfish, fish, eggs, and sesame have become the main causes of most food-related allergic reactions (Rodriguez et al., 2025) (Fig. 1). Of which milk and egg allergens are the most prominent (Gomaa & Boye, 2013), and wheat has become one of the most common food allergens after egg and milk in China (Zhang et al., 2025). The incidence of wheat allergy has been steadily increasing worldwide, and more and more people have paid attention to it. Wheat allergies can lead to a range of disorders including wheat dependent exercise induced anaphylaxis, baker's asthma, atopic dermatitis, anaphylactic shock, etc. These symptoms usually appear within minutes to hours of exposure, and the sudden whole-body reaction leads to drop in blood pressure, which in severe cases can be fatal to patient by anaphylactic shock(Du et al., 2023).

Additionally, the prevalence of wheat allergy varies from region to region and from population to population (Ricci et al., 2019). Infants are particularly susceptible to allergic reactions when exposed to food allergens, primarily due to inadequate production or secretion of digestive enzymes. However, as they mature, the development of their gastrointestinal system may alleviate these symptoms (Zhang, Shi, et al., 2025). One study found that the median age of tolerance in these patients was 7 years (3–16 years), and allergy tolerance by the ages of 8, 12, and 16 years was 52 %, 66 %, and 76 %, respectively (Czaja-Bulsa & Bulsa, 2017). In a meta-analysis of the global prevalence of wheat allergy, the global self-reported prevalence of wheat allergy was 0.63 %, with 0.58 % in children (Liu et al., 2023). Based on epidemiologic studies, the self-reported lifetime prevalence of wheat allergy in Europe was as high as 3.6 % (Nwaru et al., 2014), and researchers showed that about 0.2 %–0.5 % of children under 14 in Europe were allergic to wheat (Liu et al., 2023). Morita et al. (2012) found that the prevalence of wheat allergy in Japanese adults was 0.21 %, which was conducted by using a questionnaire, skin prick test and the serum omega-5 gliadin-specific IgE test. In another study, the Japan Environment and Children's Study (JECS) of 103,060 pregnant women and their children found that the prevalence of immediate wheat allergy reported by caregivers was 0.5 %, 0.4 %, and 0.2 % at age 1, 2, 3 years, respectively (Yamamoto-Hanada et al., 2020). Wang, Zhuang, Ma, Zhang, and Wang (2018) investigated the self-reported prevalence of food allergy in six districts in Inner Mongolia, northern China, which showed that the prevalence of food allergy was high in this region (18.0 %), with as many as 3.6 % of children showing allergic to wheat. There are many factors that contribute to these differences, including genetic background, dietary habits, and environmental factors.

Currently, an effective treatment strategy for wheat allergy patients is strict avoidance of wheat allergens (Singla, Malik, Singh, Thakur, & Kumar, 2024). With the rapid development of food industries, the incidence of food allergy is increasing year by year globally with more and more cross-contact between various food ingredients, which makes it difficult to strictly avoid the contact with allergens. Oral immunotherapy (OIT) has received research attention as an alternative to desensitization due to its ability to modify abnormal immunologic mechanism of IgE-mediated food allergy. While permanent tolerance is not often achieved, desensitization can be achieved and maintained with daily ingestion of the offending food (Pacharn & Vichyanond, 2017). However, few clinical trials have been conducted on wheat OIT, and there is safety concerns associated with OIT, with the possibility of adverse reactions in food-allergic patients during the dose escalation phase. Therefore, it is particularly important to develop hypo/non-allergenic products by modifying wheat and wheat products using different food processing methods. Nowadays, a variety of food processing technologies have been successfully applied to reduce the allergenicity of wheat products, and a range of products have emerged, such as hypoallergenic breads, noodles, cookies, pasta and sauces. The degradation of allergens in these products is mainly achieved through heat treatment, enzymatic treatment, fermentation processes or deamidation (Shukla, Gharote, & Muchahary, 2025). However, there are still important challenges to develop more diverse and safer hypoallergenic wheat food products using more efficient technologies in order to meet the growing dietary needs of wheat-allergic populations.

In this review, we provide an overview of the mechanism of wheat allergic reactions, major allergens, antigenic epitopes, and cross-allergy with other cereals. The processing methods to eliminate or reduce wheat allergens are discussed, while animal experimental models are introduced that can objectively simulate the allergic reactions induced by the food *in vivo*. This review aims to provide basic information to researchers, common man and allied stakeholders (Pasha et al., 2013), not only to provide a certain reference value for further research on wheat processing and utilization, but also to maximize the benefits of wheat as a protein source and minimizing the adverse effects of wheat allergy.

## 2 Wheat allergy

### 2.1 Mechanisms of wheat allergy

**Fig. 2:**
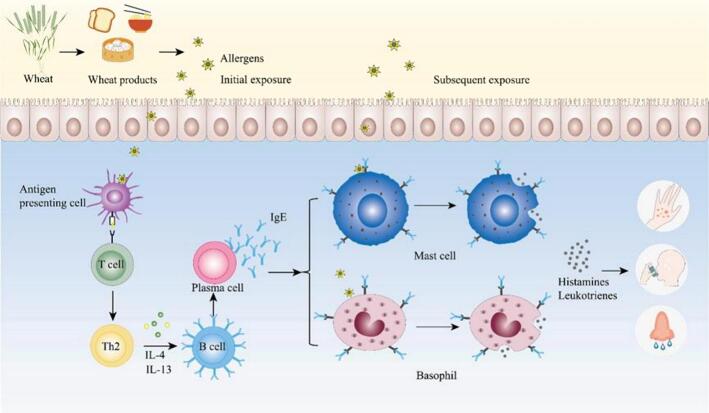
Immunoglobin E (IgE)-mediated food allergy mechanism.

The pathogenesis of wheat allergy is similar to that of other common food allergies, where the allergen is transported to immune cells, ultimately causing allergic symptoms (Liu et al., 2023). Current research suggests the following three mechanisms of reaction to food allergy: IgE-mediated food allergy, non-IgE-mediated food allergy, and food allergy mediated by a combination of the two (Santos et al., 2023). Wheat allergy is primarily a type I allergic reaction mediated by IgE (Yao et al., 2019), including three stages of sensitization, excitation and effect (Abe et al., 2020). When food allergens or allergenic fragments enter the body of allergic patients for the first time, they pass through the gastrointestinal mucosa, which are captured by antigen presenting cells and presented to T cell receptors, leading to T cell activation and differentiation into Th2 cells. The Th2 cells secrete cytokines, such as interleukin-4 (IL-4), which can induce B lymphocytes to transform into plasma cells. Plasma cells then release specific IgE antibodies, which spread throughout the body as the blood circulates, bind to mast cells and high-affinity receptors on the cell membrane of basophilic granulosa, making these cells become target cells for sensitization, making the body in the sensitization stage (Lamiable, Mayer, Munoz-Erazo, & Ronchese, 2020). When the sensitized organism is re-exposed to the allergen, it specifically binds to the IgE antibodies already bound to the target cells, inducing the cells to undergo a degranulation reaction, releasing histamine, leukotrienes, and other reactive mediators. These active mediators can cause an inflammatory response, acting on the effector tissues and organs, thereby triggering a local or systemic allergic reaction (de Jong & Wichers, 2022).(Fig. 2).

Clinical symptoms usually manifest as respiratory problems, such as conjunctivitis, rhinitis, and asthma, while skin reactions, such as hives, eczema, and itching (Lee et al., 2022). Moreover, it also causes gastrointestinal problems, such as vomiting, abdominal pain, and diarrhea as well as symptoms, such as low blood pressure and impaired thinking (Sabença et al., 2021). These symptoms usually appear within minutes to hours of exposure.

### 2.2 Types of wheat allergens

Wheat allergy is caused by a wide variety of allergens. Different wheat allergens have different pathogenic mechanisms, and allergic patients experience different clinical symptoms (Pourpak, Ghojezadeh, Mansouri, Mozaffari, & Farhoudi, 2007). Both the establishment of wheat allergen detection methods and the search for methods to reduce allergenicity need to be based on the premise of clarifying information about the major wheat allergen proteins. Based on the solubility of the proteins, wheat proteins are usually classified into three categories, namely gliadin, glutenin, and soluble proteins. Of these, gliadin accounts for about 40–50 %, glutenin for about 30–40 % and soluble proteins for about 15–20 % (Janssen, Mesure, & Wouters, 2022; Zhang, Lv, Jin, Ren, & Wang, 2023). As of September 21, 2023, WHO/IUIS Allergen Nomenclature Sub-Committee have approved 28 wheat allergens, which can be categorized into 9 foodborne allergens and 19 inhalant allergens, depending on exposure conditions. Food allergy to wheat is more common in children and is a concern because of the high risk of exposure with severe allergic reactions, and inhaled wheat allergy can trigger asthma or rhinitis in bakers, which is a common occupational disease among workers with high exposure to wheat flour, such as bakers (Cianferoni, 2016). The biochemical names, molecular weights, and allergen exposure routes of these foodborne allergens are listed in Table 2.

**Table 2:** Foodborne wheat allergens (adapted from WHO/IUIS Allergen Nomenclature Sub Committee).

Allergen	Biochemical name	Molecular Weight (SDS-PAGE)	Route of Allergen Exposure
Tri a 12	Profilin	14 kDa	Food
Tri a 17	β-amylase	56 kDa	Food
Tri a 18	Agglutinin isolectin 1		Food
Tri a 19	ω-5 gliadin	65 kDa	Food
Tri a 20	γ-gliadin	35–38 kDa	Food
Tri a 25	Thioredoxin		Food
Tri a 26	High molecular weight glutenin	88 kDa	Food
Tri a 36	Low molecular weight glutenin	40 kDa	Food
Tri a 37	α-purothionin	12 kDa	Food

#### 2.2.1 Gliadin

Gliadin exists as peptide single chains, rich in glutamine and proline, which can be classified into four isoforms, namely α, β, γ and ω; Such classification is based on their electrophoretic mobility, with sensitization present in each isoform(Lavoignat et al., 2024). They are highly fluid and help to enhance the stickiness and extensibility of the dough. Both α- and β-gliadin proteins have comparable primary structures consisting of around 250 and 300 AA residues; α-gliadin are more damaging to the mucous membranes of the intestines, showing a major allergen in wheat allergy and celiac disease (Japelj et al., 2020). Morita, Yamamura, Mihara, Kameyoshi, and Yamamoto (2000) showed that γ-gliadin is a major allergen that triggers wheat-dependent exercise-induced anaphylaxis, which also showed to be an important allergen in celiac disease. In addition, Matsuo et al. (2004) showed that ω5-gliadin is also a major allergen that triggers wheat-dependent exercise-induced anaphylaxis.

#### 2.2.2 Glutenin

Glutenin molecules are connected by disulfide bonds; their amino acids are mostly polar amino acids, which easily aggregate, giving the dough strength and elasticity (Qu et al., 2024). According to the size of relative molecular mass, they can be categorized into high-molecular-weight glutenin subunit (HMW-GS) and low-molecular-weight glutenin subunit (LMW-GS) (Mendez, Una, Vega-Fernandez, & Santos, 2022). The latter one is an important allergen in susceptible populations, which has been associated with celiac disease and wheat contact dermatitis (Wang, Tong, Zhou, Yang, & Fu, 2023). The HMW-GS and ω-5-gliadin are major allergens in wheat-dependent exercise-induced anaphylaxis (WDEIA) and their IgE-binding levels can be an important indicator for assessing WDEIA (Matsuo, Kohno, Niihara, & Morita, 2005).

#### 2.2.3 Soluble proteins

Soluble proteins nutritionally rich, which include albumin and globulin, and are structural and enzymatic proteins in cytoplasm. They include α-amylase, trypsin inhibitors (ATIs), wheat lipid transfer proteins etc., among them, α-amylase inhibitors are major allergens triggering baker's asthma, food allergy, and WDEIA (Geisslitz et al., 2021). A 36 kDa wheat glycoprotein was also reported to be an important allergen causing asthma in bakers (Tsuji, Kimoto, & Natori, 2001).

Currently, there has been some advancement in the study of wheat allergen characterization, quantification, structural identification, functional characterization, and molecular interaction processes. Allergens can be quantified and characterized for allergen or molecular interactions by enzyme-linked immunosorbent assay (ELISA), real-time polymerase chain reaction (PCR), biosensors, immunoblotting etc. (Hu et al., 2023). There are researchers who keep on identifying the wheat allergens. For example, Baar et al. (2012) screened wheat using the serum IgE from food allergy patients in a wheat cDNA expression library and discovered a new wheat allergenic protein as Tri a 36. All these studies and approaches attempt to minimize the risk of food allergy by identifying direct target proteins before people develop food allergies.

### 2.3 Epitopes of wheat allergens

Immune cells generally have difficulty in recognizing the entire antigenic molecule with the help of its surface receptor. It can only recognize a specific part of the protein antigenic molecule, and this specific part is the epitope, which can also be called the antigenic determinant cluster. It is a special chemical group in the allergenic molecule that determines the antigenic specificity (Guo & Cong, 2024). Epitopes are usually categorized into linear and conformational epitopes based on their structural features. Linear epitopes consist of contiguous amino acids, while conformational epitopes are spatial structures formed by folding of disconnected amino acid residues (Peters, Nielsen, & Sette, 2020). They can also be categorized into T-cell epitopes and B-cell epitopes according to their binding receptor cells (Zhang et al., 2023). T-cell epitopes are generally linear epitopes, while B-cell epitopes can be either linear or conformational epitopes. Unlike T-cell epitopes, B-cell epitopes can cross-link with IgE antibodies, which are directly involved in the elicitation phase of food allergy, and are closely linked to the effector phase, serving as a determinant of food allergy. Predicting and characterizing linear B-cell antigenic epitopes can help to further understand the occurrence of food allergic reactions and their epitope changes during processing.

The existence of antigenic epitopes on the protein structure is more complex. It is difficult to locate and predict them by using traditional single experimental methods, while it has the disadvantages of complicated operation and low efficiency. With the development of bioinformatics, the antigenic epitopes of allergens can be obtained quickly, accurately and efficiently by using bioinformatics databases and related software (Lin, Chi, Ni, Zhang, & Liu, 2023). Table 3 lists common B-cell epitopes prediction tools and prediction principles. Moreover, bioinformatics can provide three-dimensional models of allergen structures, which can help to visualize the distribution of linear and conformational epitopes on the surface of proteins. In addition, bioinformatics can be used to validate the results of traditional strategies to ensure the reliability of the conclusions, which can be an effective means of assessing the potential sensitization of unknown allergens (Zhou, He, Sun, Wang, & Zhang, 2021).

**Table 3:** List of web available tools for linear B-cell epitopes prediction.

Methods	Websites	Predictive principles
BepiPred	http://www.cbs.dtu.dk/services/BepiPred/	Hidden Markov models and hydrophilicity parameter scores
ABCpred	https://webs.iiitd.edu.in/raghava/abcpred/ABC_submission.html	Recurrent ANNs, fixed length epitope patterns
BcePred	https://webs.iiitd.edu.in/raghava/bcepred/bcepred_submission.html	Physico-chemical properties
AAPPred	https://www.bioinf.ru/aappred/	Amino acid pair antigenicity scale
COBEpro	http://scratch.proteomics.ics.uci.edu/	SVM, epitopic propensity score, secondary structure, solvent accessibility information
IEDB	http://tools. Immuneepitope.org/ bcell/	A collection of tools based on various methods
SVMTriP	http://sysbio. unl.edu /SVMTriP/	Tri-peptide similarity, Propensity scores
LBtope	https://webs.iiitd.edu.in/raghava/lbtope/	Large datasets of epitopes, SVM

Several tools have been used to predict antigenic epitopes, such as DNAstar, Bepibred (Bu, Li, Zhu, & Xi, 2020), ABCpred (Anandhan, Narkhede, Mohan, & Premasudha, 2023), BcePred, IMED, IEDB, SVMTriP (Yue et al., 2025) etc. However, all prediction methods have some limitations in their use; therefore, a combination of methods is usually used for epitope prediction. Finally, based on the predicted epitopes, 3D models can be created using PyMoL or SWISS-MODEL to visualize the epitopes. For example, Wang, Zhang, Wang, and Fu (2023) predicted the linear epitopes of wheat non-gluten sensitizer α-amylase inhibitor by bioinformatics prediction tools, namely DNAstar, IMED, and IEDB. A comprehensive analysis yielded six candidate epitopes, which were validated using indirect competition enzyme-linked immunosorbent assay. A comprehensive analysis yielded the amino acid at 5–12, 27–31, 57–81, 96–108 region as its potential epitopes. However, there are fewer studies on wheat allergen antigenic epitopes; so, it is of great significance to predict wheat allergenic antigenic epitopes by bioinformatics. It is still under study to provide theories and methods for predicting antigenic epitopes of different food allergenic proteins.

### 2.4 Cross-allergy

Food cross-allergy refers to the susceptibility to cross-reactivity when two proteins are highly homologous in sequence or structure, with an increased likelihood of identical antigenic determinants(Faihs, Kugler, Scherf, Biedermann, & Brockow, 2024). Jones, Magnolfi, Cooke, and Sampson (1995) reported that 20 % of grain-allergic patients react to more than one grain. Some wheat-allergic patients may develop allergic symptoms to other grains, while others may be able to consume these grains without allergic symptoms. Therefore, cross-reactivity between wheat and other cereals, especially barley, rye, oats, and other plants in the same family of grasses is a key issue in the management of wheat allergy.

In children with immediate-type wheat allergies, Yanagida, Takei, Saito, Sato, and Ebisawa (2022) confirmed that half of the patients with wheat allergies reacted to barley, demonstrating a clinical cross-reactivity between the two grains. The reactivity to barley was associated with higher wheat-specific IgE levels and lower wheat threshold doses, indicating that wheat was the primary allergenic food for patients, barley causes secondary sensitization due to cross-reactivity to wheat. In order to detect the cross-reactivity among wheat, barley and rye, Takei, Saito, Yanagida, Sato, and Ebisawa (2022) collected sera from 128 children with immediate wheat allergy and determined the specific immunoglobulin E (sIgE) levels of the fractions of wheat, barley and rye by ELISA techniques. The results showed that sensitization to wheat was higher than that to barley and rye, indicating that wheat was the primary allergen source, while the sensitization to barley and rye was caused by cross-reactivity. Zhao et al. (2021) found that high cross-reactivity because of high sequence similarity (>50 %) between wheat and barley and rye, whereas cross-reactivity with rice, buckwheat, and quinoa was almost negligible. Therefore, for wheat allergy sufferers, rice, quinoa, and non-grain buckwheat can serve as substitutes for wheat in their daily diet, while barley and rye should be avoided.

The high degree of similarity in protein sequences and structures is the main process of cross-reactivity between different graminaceous plants. Therefore, consumers who are allergic to wheat should also be careful when consuming other graminaceous plants. However, not all graminaceous plants inevitably cross-react with wheat, and not all people are allergic to a wide range of graminaceous plants; however in reality, it is difficult for allergic consumers to exclude any graminaceous plants from their diets. So, there is an urgent need to provide wheat-allergic consumers with accurate guidance for avoiding allergens that may be a health crisis, and there is a need for allergen detection and identification More research is needed to better achieve this purpose.

## 3 Technologies to reduce wheat allergenicity

**Fig. 3:**
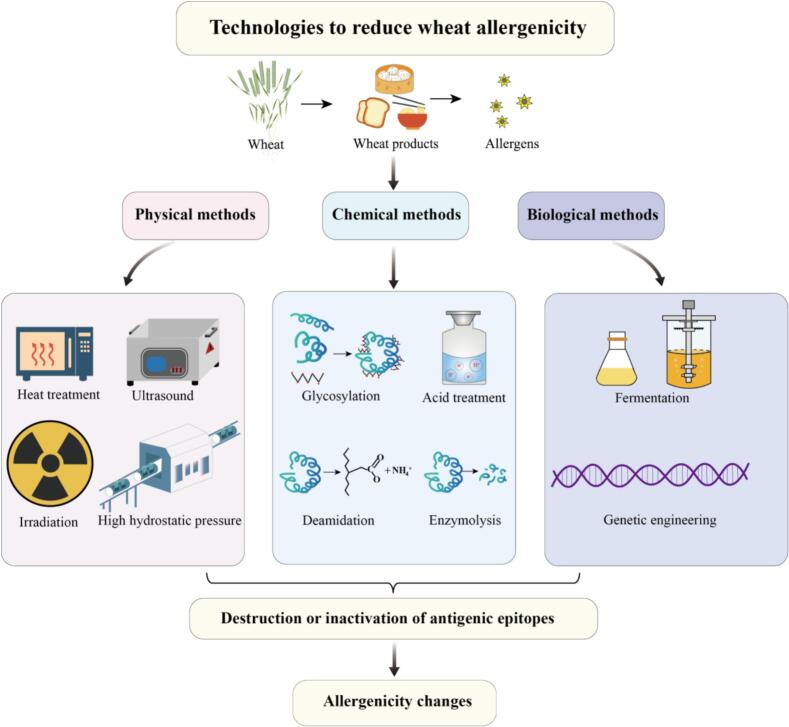
Technologies to reduce wheat allergenicity.

The essence of sensitization reduction or elimination of wheat allergens is the destruction or inactivation of their antigenic epitopes (Cuadrado et al., 2023). The purpose of food processing is to improve the functional, nutritional and sensory qualities of foods in order to ensure for safe consumption. It is one of the most important aspects in the field of food safety. Complex food processing can have different degrees of influence on the structure and sensitization of food allergenic proteins. Based on this, the allergenicity of foods can be changed to certain extent by processing methods (Kang, Zhang, Yu, He, & Chen, 2023). How to reduce allergenicity while ensuring the sensory and quality of food has become a major concern. According to different principles, the relevant processing methods can be classified into physical, chemical, and biological methods (Fig. 3). Table 4 also lists the advantages and disadvantages of different processing methods.

**Table 4:** Advantages and disadvantages of different processing methods in reducing allergenicity.

Processing methods	Advantages	Disadvantages
Heat treatment	Simple, convenient, and low-cost	New antigenic epitopes may be generated and nutrient loss, not applicable to allergens with high heat resistance
Ultrasound treatment	Simple, safe, environmental-friendly, and low-cost	May destroy nutrients and increase allergenicity
Irradiation treatment	High energy penetration, large processing capacity, and high efficiency	Potential safety hazards and nutrient loss
High hydrostatic pressure	High efficiency, and maximize the retention of nutrients in foods	Processing conditions need to be further explored
Glycosylation	Maintaining nutritional and functional properties	Reaction conditions are complex
Deamidation	Changing physical and chemical properties, and enriching products	May generate new allergens, change the physicochemical properties of wheat products
Acid treatment	Simple, low cost, and suitable for large-scale industrial applications	Acidolysis can produce a bitter taste, some safety risk, may affect food quality
Enzymatic treatment	Fast, mild, and high efficiency	Influenced by other conditions, produce by-products
Fermentation treatment	Efficiency and not destroy other nutrients	Limited scope of application and subject to conditions
Genetic engineering	Precision, standardization, and high productivity	High cost, complex, security needs to be considered

### 3.1 Physical methods

Physical sensitization of wheat allergens uses physical approaches, including heat, electricity, magnetism and mechanical energy. Physical methods usually lead to intermolecular aggregation of allergens, which alters the high-level structure of proteins and reduces the allergenicity of foods to some extent (Fu, Cherayil, Shi, Wang, & Zhu, 2019). Physical modification has the advantages of low cost, no toxic side effects, short action time, and little impact on the nutritional characteristics of processed products (Yang et al., 2023). Commonly include heat treatment, ultrasound, irradiation, and high hydrostatic pressure.

#### 3.1.1 Heat treatment

Heat treatment leads to loss of protein secondary and tertiary structure as well as non-covalent interactions that reduce its binding activity to human IgE with an aim to eliminate their allergenicity. Heat treatment reduces the allergenicity of wheat, peanuts, milk, eggs, etc., but crustaceans and some tree nuts are highly resistant to heat (Cabanillas & Novak, 2017). Lupi et al. (2019) showed that heat-treated gliadin existed as aggregates, allowing more antigenic epitopes of gliadin to be hidden with no longer recognized by IgE in the patient's serum, which showed the reduction of allergen sensitization. Kwak et al. (2011) treated gliadin with high-pressure, microwave, and combined high-pressure-microwave methods. Among them, a high-pressure steam cooker for 50 min resulted the degree of binding of gliadin to the antibodies decreased to about 69 %, while after autoclaving for 50 min and microwave treatment for 5 min, the binding degree decreased to about 73 %, which was not significantly different from that of the autoclave treatment alone, while the microwave treatment alone did not have an effect. Some researchers have detected gluten allergens in baked cookies by ELISA and flow cytometry and found that allergen recovery decreased with increasing heat treatment time, which was associated with heating changing the allergen structure and the occurrence of the Maillard reaction to change allergen epitopes (Gomaa & Boye, 2013). Cooked pasta was reported losing half of the allergenicity of extractable non-gluten proteins (Gao et al., 2021). In addition, pasta allergenicity is affected by the screw speed during extrusion, and Jia et al. (2025) found that a screw speed of 550 rpm resulted in the loss of allergenic epitopes and a reduction in the number of allergens in pasta after *in vitro* digestion, leading to decreased sensitization.

Heat treatment is widely used to reduce the allergenicity of wheat proteins to some extent. However, heat treatment may also lead to a creation of new epitopes, increasing their ability to bind with IgE, thereby increasing food allergenicity (Pi, Zhu, Liu, & Zhang, 2024). At extreme temperatures, the nutrient content of the food may also be negatively affected.

#### 3.1.2 Ultrasound treatment

Ultrasound refers to sound waves with frequencies ranging from 20 kHz to 1 GHz, with the most commonly used range in food processing being 20–30 kHz. (Huang et al., 2020). Ultrasound is capable of producing thermal, mechanical and cavity effects that can degrade proteins or have an effect on their spatial structure(Pang et al., 2024). It is often used alone to reduce allergenicity in foods including milk, shellfish, wheat, soy, eggs, nuts, fish, etc. (Pi et al., 2024). Ultrasound treatment had no effect on the protein electrophoretic patterns of gliadin and altered the secondary and tertiary structure, destroying and masking conformational and linear epitopes, thereby reducing allergenicity. In addition, temperature was found to significantly influence the efficacy of ultrasound treatment in reducing allergens. Li, Linhong, and Jamil (2006) treated *Penaeus vannamei* with high-intensity ultrasound (30 Hz, 800 W) at 0 °C and 50 °C for 1.5 h. The results indicated that 50 °C was the most effective in reducing allergenicity, while there was almost no effect at 0 °C. This might be because the allergenic substances absorbed the energy of ultrasound and converted it into thermal energy and such thermal energy degraded the allergenic substances.

#### 3.1.3 Irradiation treatment

Irradiation promotes the oxidation of proteins, causing them to break down and aggregate. Radiation desensitization is caused by the food matrix directly absorbing energy from γ-rays, x-rays, or electron beams. This alters the spatial structure and conformation of the sensitized protein and eliminates the epitope of the sensitized protein, thereby reducing the degree of sensitization of the protein. To date, only a few studies have reported on the use of irradiation in reduction of wheat allergens, mainly in the desensitization of soybeans, peanuts, milk, eggs, and shrimp (Pan et al., 2021). Vaz et al. (2012) treated an agglutinin (WGA) with γ-radiation (1, 10 and 25 kGy) and found that irradiation doses of 10 kGy or more reduced WGA sensitization because gliadin is a hetero agglutinin in wheat, thus confirming the ability of γ-radiation to reduce allergenicity in wheat. Luo et al. (2013) isolated peanut allergen (Ara h 6); both the allergens and whole peanut protein extract (WPPE) were irradiated at 1, 3, 5 or 10 kGy. As the irradiation dose increased, the secondary and tertiary structures of Ara h 6 changed and the antigenicity of both purified Ara h 6 and WPPE decreased.

Irradiation technology, as an emerging non-thermal physical treatment has high treatment efficiency. However, it is not widely accepted in the market at present because the effect of strong irradiation on allergic reactivity is complex with possible dose residues of the irradiation intensity.

#### 3.1.4 High hydrostatic pressure

High hydrostatic pressure (HHP) technology effectively kills microorganisms and inactivates enzymes, and also maximizes the retention of nutrients in foods, while it has shown potential in reducing food protein allergenicity. High hydrostatic pressure treatment deals with the secondary and tertiary structure of proteins to produce a certain degree of damage, thereby destroying the conformational epitope of protein allergens in order to achieve the effect of reducing allergies or desensitization (Gharbi, Marciniak, & Doyen, 2022). Recent studies have increasingly reported the use of HHP in reducing food allergenicity, especially in common allergen sources such as wheat, milk, nuts and aquatic products (Zhang et al., 2025). Bu, Li, Zhao, and Chen (2020) showed that the binding ability of HHP-treated β-conglycinin to epitope antibodies was inhibited, compared to the untreated group. Yao, Jia, Lu, and Li (2022) investigated the effects of high hydrostatic pressure (200, 300, 400 and 500 MPa), treatment time (5, 10, 15, 20 and 25 min), and protein concentration (1, 3 and 5 %) on the structure of wheat gluten sensitization. The results demonstrated that wheat gluten sensitization was reduced by 72.2 % under the combination of 400 MPa, 20 min treatment and 3 % protein. However, further validation by *in vivo* studies was recommended.

High hydrostatic pressure has received increasing attention for its ability to effectively control food safety and quality, while avoiding the inevitable harmful side effects of high temperatures. Therefore, it is of great value to study the alteration of wheat allergenicity by high hydrostatic pressure to produce high quality wheat products with sensory, nutritional and hypoallergenic characteristics.

### 3.2 Chemical methods

Chemical methods involve chemically altering the structure, electrostatic charge, and hydrophobic groups of proteins to improve the functional properties of proteins, which can hide or disrupt the sensitizing epitopes of the allergenic protein molecules, thereby altering their allergenicity. Compared with other treatments, chemical modification methods have many advantages, including short reaction time, low cost, no need for specialized equipment, and very obvious modification effects (Abedi & Pourmohammadi, 2021). Commonly used chemical methods include glycosylation modification, deamidation modification, acid-base treatment, and enzymatic treatment.

#### 3.2.1 Glycosylation

Glycosylation can affect the immunogenicity of food allergenic proteins by allowing them to bind to glycans via a Maillard reaction that masks their antigenic epitopes. The Maillard reaction is one of the pathways for the glycation of food proteins and is a common non-enzymatic browning reaction in food processing. It mostly takes place between the amino group of proteins and the carbonyl group of reducing sugars. This method is not only able to shield or bury some of the sensitizing epitopes of proteins, but also able to reduce the antigenicity and allergenicity of proteins. It does not require the addition of any chemicals. However, different reducing sugars will have different degrees of influence on the protein properties after glycosylation modification (Ma et al., 2021).

Glycosylation modification can effectively reduce the allergenicity of a variety of allergens, such as tropomyosin, whey allergens, chickpea albumin (Zhou, Li, Zhu, Chen, & Wu, 2024). In addition, it also has a reducing effect on the allergenicity of allergenic proteins in wheat and buckwheat. Hou et al. (2024) used monosaccharides (glucose, fructose, and galactose) to prepare complexes by glycosylation reaction with wheat sensitizing proteins and analyzed the difference in the sensitization of wheat sensitizing proteins before and after the glycosylation reaction by using SDS-PAGE, ELISA and Western blot. The experimental results showed that glycosylation modification reduced the sensitization of wheat gluten proteins with a stronger effect on the sensitization of gliadin than on glutenin. Yang, Li, Li, and Wang (2013) prepared polysaccharides from buckwheat flour, which after heating, were covalently bound to Fag t 3 via a Maillard reaction. The binding properties of Fag t 3 to IgE/IgG were significantly reduced.

Although glycosylation reactions have a greater potential to reduce allergenicity, the application of current methods in food industries is limited by the uncontrollable nature of chemical reaction, which may result in the formation of anti-nutrient-like substances during the reaction process.

#### 3.2.2 Deamidation

Deamidation is a reaction, in which the side chain amide of a protein is deamidated to a carboxyl group, disrupting the conformation of the protein and thus weakening its sensitizing properties. Previous studies have successfully used deamidation to alter the sequence of wheat gliadin and reduce wheat allergenicity (Liu, Dong, Yin, Zhang, & Jia, 2025). Kumagai et al. (2007) found that deamidation of gliadin reduced its reactivity towards the sera of patients with wheat allergy. After injecting deamidated gliadin into rats, the increase in gliadin-specific IgE levels was suppressed. However, some studies have shown that deamidation increases allergenicity. For example, Gourbeyre et al. (2012) compared the allergenic and inducible potentials of natural gliadin (NG) and deamidated gliadin (DG); the results indicated that DG has a higher sensitization potential than NG. In addition, deamidation of wheat gluten by citric acid results in unfolding of the gluten structure and reduction of allergenic epitopes in the 35–63 kDa region, which reduces sensitization, and that 25 % degree of deamidation can be used to make hypoallergenic noodles, and the addition of azodicarbonamide improves the texture of the noodles (Liu, Dai, Yin, Huang, & Jia, 2023).

It is evident that deamidation modification can change the physicochemical properties of wheat products, which may also increase sensitization, making it difficult to ensure the absolute safety of the food. In conclusion, deamidation is a double-edged sword; therefore, it is not yet a better method for sensitization in wheat allergens.

#### 3.2.3 Acid treatment

Acid treatment of allergenic proteins causes cleavage of the proteins, triggers changes in the polarity of the protein surface, and the formation of insoluble complexes, which lead to conformational changes in the allergenic proteins, affecting the ability of the allergen to bind specifically to IgE, thus reducing or eliminating the sensitizing properties of the allergens. By summarizing the cases related to the reduction of allergens by acid treatments, researchers have found that acid treatments are effective in reducing the allergenicity of foods such as eggs, chicken and lentils (Zhu, Vanga, Wang, & Raghavan, 2018). Additionally, studies have shown that acid treatments have a similar effect on the sensitization of allergens such as wheat and peanuts. Maruyama et al. (1999) treated gluten proteins with lactic acid and hydrochloric acid; at 50 % deamidation, gluten proteins showed a significant decrease in their ability to bind IgE. Kim et al. (2012) found a significant decrease in allergen sensitization after soaking peanut allergens (Ara h 1, Ara h 2, and Ara h 3) using an acetic acid solution at pH 1.0. Chung and Reed (2012) found that when peanut allergens were mixed with 0.5, 1 and 2 mg/mL of tannic acid, insoluble complexes were formed; hen these complexes were eliminated, the binding capacity of the extracts to their IgE was significantly reduced by approximately 55 %, 75 %, and 100 %, respectively.

Although acidolysis can reduce the allergenicity of wheat, acidolysis can produce a bitter taste, while the use of hydrochloric acid for food ingredient treatment has been limited in practical applications. Therefore, the search for an edible organic acid to reduce the allergenicity of wheat has more important significance.

#### 3.2.4 Enzymatic treatment

Enzymatic methods are mainly used to reduce the allergenicity by partially or completely hydrolyzing allergenic proteins into peptides or amino acids, which further destroys the linear and conformational epitopes of allergens. Some studies have shown that specific allergy-related proteins of allergens such as wheat, soybean, and kiwifruit are hydrolyzed by related enzymes (Wang, Vanga, McCusker, & Raghavan, 2019). Lu, Ouyang, Liu, Liu, and Li (2017) screened bromelain and papain to effectively reduce the allergenicity of wheat flour. They also adopted the simultaneous method of enzyme digestion and obtained the optimal enzyme digestion conditions through response surface analysis as material-liquid ratio of 6.64 %, enzyme digestion pH of 6.53, enzyme digestion temperature of 47.7 °C, enzyme digestion time of 2.47 h, and the amount of enzyme addition of 0.395/100 g of protein. The study showed that the allergenicity was significantly reduced by 36.1 % under this condition. Li, Yu, Goktepe, and Ahmedna (2016) selected effective enzymes to catalyze the hydrolysis of gliadin in wheat flour by testing six proteases and showed that alcalase and papain treatment of wheat significantly reduced the *in vitro* allergenicity of wheat flour. There is evidence that treatment of flour with actinase and cellulase can produce hypoallergenic wheat flour that can be used to make bread and cupcake products (Tanabe, 2008). By consuming hypoallergenic cupcakes over a long period, more than half of patients become hypoallergenic and can eat normal wheat products. Hypoallergenic wheat flour may exert an anti-allergic effect through allergen-specific immune tolerance.

Compared with other chemical methods, the enzymatic method has the advantages of high efficiency, mild reaction conditions, low toxicity and side effects, without diminishing the nutritional value of proteins in foods. The enzymatic treatment outcome varies substantially at different temperatures.

### 3.3 Biological methods

Biological methods mainly include fermentation technology and genetic engineering, which directly disrupt the peptide chain structure of allergenic proteins. Such allergen inhibition is extremely effective; however, it is more costly and destructive to proteins.

#### 3.3.1 Fermentation treatment

Fermentation is the process of preparing microbial cells or secondary metabolites through microbial life activities under aerobic or anaerobic conditions (Kumar et al., 2021). During fermentation, microorganisms secrete a certain amount of proteases and accompany the onset of hydrolysis, which disrupts their linear and conformational epitopes, inhibiting specific antibody-specific recognition. Fermentation treatment can be used as a new technology for food sensitization process because it does not destroy other nutrients, compared to the methods of heat treatment and chemical processing. In recent years, fermentation has been widely used to reduce the allergenicity of soybeans, milk, wheat, peanuts, aquatic products, etc. (Zeng et al., 2024).

Phromraksa, Nagano, Boonmars, and Kamboonruang (2008) found that the protein-hydrolyzing bacteria isolated from traditional Thai fermented foods were *Bacillus subtilis*, while *Bacillus subtilis* DB and SR reduced the sensitization of gliadin. Leszczynska et al. (2009) found the greatest decrease in the immunoreactivity of the maltolysin fraction when *Lactobacillus* and yeast were fermented in a mixed culture, suggesting a synergistic effect of the two microorganisms in decreasing the immunogenicity of the gliadin. Furthermore, it has been demonstrated that yeast and probiotics have the advantage of lessening food allergenicity. In addition to breaking down allergenic epitopes, they also generate oligopeptides that have stronger antibacterial, anti-inflammatory, and antioxidant properties and can alter the flavor of food (Nath et al., 2021). The Italian company Giuliani (Milan, Italy) produces a gluten-free wheat bread called “Giusto Sapori Tradizionali Bontà di Pane”, made from special sourdough-fermented wheat flour. During the 24 h of yeast fermentation, fungal peptidases degrade the proteins into oligopeptides, which are then hydrolyzed to amino acids by a patented LAB blend (Scherf, Wieser, & Koehler, 2018). This product is particularly important in terms of nutrition for celiac disease and wheat allergy sufferers, being hypoallergenic and offering improved flavor, texture and good nutritional properties. However, fermentation is affected by several conditions, such as microbial species, temperature, pH and substrate concentration; so, the effect is not stable and needs further in-depth study.

#### 3.3.2 Genetic engineering

The principle of genetic engineering to reduce the allergenicity of food allergens is mainly through recombination, knocking out genes at specific loci or hindering the expression of allergenic proteins, so that allergenic sequences cannot be expressed normally. Sánchez-León et al. (2017) designed two small guide RNAs to target a conserved area near the 33-mer coding sequence in α-wheat glycolysin gene. It was shown that the α-wheat glycolysin content was significantly reduced in all lines, while the immunogenicity of the modified wheat strains decreased by 85 %. Waga and Skoczowski (2013) utilized traditional plant breeding methods to obtained a set of wheat genotypes lacking all ω-gliadins by accumulating inactivated gene variants at three gliadin coding loci (*Gli A1*, *Gli B1*, and *Gli D1*). Comparing the endosperm proteins of ω-gliadin-free genotypes with control genotypes containing all ω-gliadins by A-PAGE, SDS-PAGE, and RP-HPLC; by using sera from 10 patients with gluten sensitivity, ELISA confirmed a significant reduction in gliadin immunoreactivity (about 30 %).

Currently, genetic engineering has been reported to edit allergen genes in wheat, soybeans, peanuts, and brown mustard to reduce allergenicity (Chakraborty & Wylie, 2024), but genetic engineering desensitization is not widely used because of other unknown effects that genetic engineering may have on proteins, such as there may be problems with gene mutation or post-gene expression.

## 4 *In vivo* mouse model evaluation study of wheat products

Animal models can objectively simulate food-induced allergic reactions *in vivo*, and assess the sensitizing ability of food allergens by measuring changes in cytokines and specific antibodies and clinical symptoms in animals (Li, Bu, Chen, Zhao, & Chang, 2024). Compared to *in vitro* testing, animal testing is closer to the reality of the immune response triggered by food allergens *in vivo*. A variety of animals, such as rats, mice, pigs, and dogs have been used as research subjects; however, despite the value of large animals for the study of food allergy, mouse models have become the preferred choice for research because of their small size, short reproduction cycle, immunological characteristics, and ease of handling (Huang, Wang, Xiang, & Zheng, 2014). In addition, the genetic characteristics of mouse strains are one of the key factors influencing the development of food allergy, and common mouse models include BALB/c, C57BL/6, C3H/HeJ, A/J and KM (Zhou et al., 2024). Among them, BALB/c mice are a commonly used animal test model for food allergy, which are widely used in sensitization assessment studies (Gouel-Chéron, Dejoux, Lamanna, & Bruhns, 2023). They are dominated by Th2-type cellular responses and are more susceptible to allergens, which are capable of producing highly efficient IgE (Yu et al., 2024). Bodinier et al. (2009) compared the sensitization of gliadin extracts in different mouse strains (BALB/c, B10.A, and C3H/HeJ), and found that BALB/c mice exhibited the most severe allergic reactions in terms of blood and spleen (IgE, IL-4/IL-5) as well as in the airways. The other two species of mice did not show allergic symptoms, suggesting that mouse allergic responses are genetically controlled. It has been shown that females are more immunoreactive than males (Wang et al., 2021), Fu et al. (2019) successfully established an allergic model of female and male BALB/c mice by intraperitoneal injection using promyosin as an allergen. The results showed that the serum levels of sIgE antibodies, histamine levels, and the production of inflammatory cytokines in the female mice exhibited a more significant response than those in the male mice.

Studies on the pathogenesis of wheat allergy in the BALB/c mouse allergy model usually use intraperitoneal injection, transdermal sensitization, and gavage sensitization to establish a sensitization model. Liu, Chen, et al. (2023) used gluten proteins combined with aluminum hydroxide adjuvant to sensitize BALB/c mice by three routes, namely intraperitoneal injection, transdermal sensitization, and gavage sensitization. The findings indicated that all three sensitization methods produced allergic reactions and increased serum antibody (total immunoglobulin E (IgE), specific IgE, IgG) and histamine levels. They also suppressed the synthesis of Th1 cytokines (IFN-γ, IL-2) and increased the secretion of Th2 cytokines (interleukin (IL)-4, IL-5, and IL-13) and inflammatory factors (IL-6, IL-17a, and IL-10). However, the most pronounced allergy reactions were displayed by sensitized mice injected intraperitoneally in the three models. Serum antibody levels were significantly higher in intraperitoneal injection group than in the control group. However, the test findings were also significantly impacted by whether an adjuvant was used or not in the intraperitoneal injection allergen test (Jin et al., 2020). Given that food allergic reactions are primarily caused by the ingestion of digested and processed foods by allergic individuals, oral gavage has become a commonly used method of food stimulation, and this method is also considered to be an effective way of assessing food triggering an allergic reaction in the body.

Animal models are effective testing methods for preclinical applications of drugs and the development of hypoallergenic wheat products. Validated animal models help to advance the basic and applied research on wheat allergens, thereby contributing to the development of effective prevention and control strategies for wheat allergen.

## 5 Conclusion

Wheat is one of the world's major food crops, with an expanding demand for wheat consumption. However, wheat allergy is of great concern. There are also some patients with cross allergies to wheat and other graminaceous plants. Both the wheat allergy and cross-allergy to the allergic population of the diet show a great deal of trouble. Therefore, it is particularly important to reduce wheat allergenicity and produce hypoallergenic wheat products. Currently, it has been demonstrated that the allergenicity of wheat proteins can be effectively reduced by various processing modification techniques such as physical, chemical and biological methods, however, the immune response to protein modification is still unclear, which needs to be explored in more in-depth studies with the help of animal models and clinical validation. With the advancement of molecular biology technology, it has become possible to reduce wheat allergenicity from the genetic nature by targeting knockout or silencing of allergen-encoding genes using gene editing techniques such as CRISPR/Cas9, but this strategy faces technical challenges such as off-target effects. It is worth noting that, in the pursuit of reducing allergenicity at the same time, must take into account the nutritional value and sensory properties of wheat products, which requires the optimization of processing technology and new product development process, the establishment of a scientific quality evaluation system, to achieve the effect of desensitization and nutritional quality of the synergistic enhancement. Future research should focus on elucidating the epitope characterization of wheat allergens and their interaction mechanisms with the immune system to provide a theoretical basis for the development of safe and effective hypoallergenic wheat products.

## CRediT authorship contribution statement

**Nan Jiang:** Writing – original draft, Visualization, Software. **Yu Wang:** Writing – original draft, Methodology, Conceptualization. **Yasai Sun:** Writing – review & editing, Methodology. **Zhe Gao:** Supervision, Resources, Project administration. **Dongcheng Liu:** Supervision, Resources, Funding acquisition. **Bimal Chitrakar:** Writing – review & editing, Project administration.

## Declaration of competing interest

The authors declare that they have no known competing financial interests or personal relationships that could have appeared to influence the work reported in this paper.

## Data Availability

No data was used for the research described in the article.
